# A Case of Stone in the Bladder

**Published:** 1880-06

**Authors:** A. B. Copeland

**Affiliations:** Hamilton, Ga.


ATLANTA

Medical and Surgical Journal.

Vol. XVIII.] JUNE—1880. [No. 3.

### Original Communications.

### A CASE OF STONE IN THE BLADDER.

By A. B. COPELAND, M.D., Hamilton, Ga.

In presenting this case for publication, the writer does not bring forward anything new, nor does he expect to enlighten the profession in regard to the means of relief.

The subject, like many others affected with serious disease, had been made the dupe of the charlatan, greatly to his injury, and, indeed, life was seriously jeoparded by promises of relief without an operation with the knife. The dissolver came in his way and would gladly save him the torture of a surgeon's cutting, by imperceptibly removing the stone with never-failing solvents.

Previously to this experiment from error in diagnosis, the various plans for relief of stricure had been imposed upon him, of course with no other effect than adding to the existing irritation.

When consulted in regard to the case, I found the man emaciated, pale and feeble from the long continued suffering, and as removal of the stone was thought to be safe, even in his weak condition, it was determined upon, and the time set for the twenty-first of April. Of course the few days he was in my charge before the operation allowed little, but some improvement in his condition by the liberal use of anodynes, stimulants, nourishing food, etc.

Assisted by Drs. Mitchell, Riley and Bruce, the operation was made at the appointed time, in the usual way by left lateral incision. We had no reasons to suspect a very large stone, yet found great difficulty in the removal of one the size of a turkey's egg. The opening made would not allow its removal and crushing became indispensable to success. Though hollow in the centre, and made of concentric layers of different degrees of firmness, much force was found necessary to break down the immense calcarious mass. Besides the large size, another difficulty in the crushing and removal was found in the firm cyst which was thrown nearly entirely around it. All difficulties were, however, overcome as speedily as possible, and the fragments removed, which aggregated four ounces in weight. The stone consisted of three regular layers of different consistence and color. The resemblance to the make up of a peach was very striking. The centre was soft, friable and of a light gray color; the next, or second layer, was very hard, and of dark gray color; and the outer stratum, which, though quite hard and of ivory color, answers in the comparison to the fleshy portion of the peach. This layer is three-fourths of an inch thick, and proved a source of difficulty in the operation of crushing.

Though no analysis has been had of the stone it would seem from the different color and consistence of these layers, that they consist of different salts probably, and that the diathesis of the patient's constitution had changed during the period in which the calculus was forming. After removal of the fragments by forceps and thorough washing out with carbolized tepid water, the antiseptic dressing and daily washing out of the bladder were carried out.

Whether the theory of bacteria be true or not, there is no doubt that irritation, inflammation and putrid or septic conditions are to a large extent prevented by the carbolic acid. While some are satisfied with a knowledge of the simple practical fact that carbolic acid will arrest inflammation in a part and prevent it in injuries where it might otherwise occur, there are those who theorise themselves into the opinion that microscopic animals come in contact with denuded parts and produce these conditions.

The recovery of the case was speedy and perfect, notwithstanding the anæmic, debilitated condition at the time of operating, so that in one month the man was able to attend his ordinary labor in the farm.

